# Health-related quality of life in parents of adolescents in 2019, 2021 and 2023

**DOI:** 10.1186/s12887-026-07115-8

**Published:** 2026-06-15

**Authors:** Gudrun Rohde, Kristin Haraldstad, Sølvi Helseth, Milada Hagen, Siv Skarstein, Hilde Timenes Mikkelsen

**Affiliations:** 1 Department of Health and Nursing, Faculty of Health and Sport Sciences, University of Agder, Postbox 422, Kristiansand, 4604 Norway; 2 Department of Nursing and Health Promotion, Faculty of Health Sciences, Oslo Metropolitan University, Oslo, Norway

**Keywords:** HRQOL, parents of adolescents, socio-economic, longitudinal

## Abstract

**Purpose:**

The objective of the present study is to describe levels of HRQOL in parents of adolescents over a span of four years (2019, 2021, and 2023) and identify which of selected sociodemographic variables associated with HRQOL over time. In addition, we compared all HRQOL domains with norm data from the general Norwegian population.

**Methods:**

A longitudinal study involving 556 parents (at baseline) of adolescents from the general Norwegian population was conducted. Data were collected at baseline in 2019, in 2021 (when the COVID-19 pandemic was ongoing) and in 2023. HRQOL was assessed using RAND-36. Data were analysed using independent samples t-tests and linear mixed model for repeated measures.

**Results:**

During the four-year (2019–2023), we observed a small but statistically significant decline in most HRQOL domains, and no domains increased again after the COVID-19 pandemic. Higher education, being in paid work and having a high household income were the most important associates of high HRQOL scores. We identified some statistical differences in HRQOL between the parents in the current study and Norwegian norms at all time points, although these were of limited clinical relevance.

**Conclusion:**

Over four years, parents of adolescents experienced a small but persistent decline in HRQOL from before the COVID-19 pandemic through 2023, particularly in mental health domains, with no full return to pre-pandemic levels. Higher education, household income, and paid employment were consistently associated with better HRQOL.

**Supplementary Information:**

The online version contains supplementary material available at 10.1186/s12887-026-07115-8.

## Introduction

Quality of life and health-related quality of life (HRQOL) is increasingly used as a goal in political programs, research settings (including clinical practice) and to assess trends in the general population. In a health-promotion context, HRQOL is defined as a multidimensional construct that includes an individual’s subjective perspective on the physical, psychological, social and functional aspects of health [1]. In prior studies concerning the general adult population, demographic characteristics such as being male, achieving a higher level of education [2–4] belonging to a more affluent socio-economic stratum, having a high income, being married or cohabitating, and being in employment [2, 4, 5], have been linked to higher HRQOL. However, most of these studies are cross-sectional and longitudinal data regarding HRQOL in the general population remain scarce.

For many adults, their parental role is a crucial aspect of their lives. The experience of parenting an adolescent may involve challenges, and this parental role might exert an influence on HRQOL, with variations upon the age of their child [6–9]. Adolescence is delineated by substantial transformations on physical, cognitive and psychosocial fronts, which are connected to self-identity, peer affiliations, the evolution of autonomy and sexuality [10–12]. As adolescents’ aspirations for autonomy and independence intensify, they may manifest some resistance to familial norms and roles, leading to an amplification in parent–adolescent disputes. Furthermore, within Western cultures, the transition to adulthood in parent–adolescent relationships are perceived as facilitating adolescents’ efforts to distinguish themselves from their parents and become more independent [13, 14]. For most adolescents, their relationship with their parents tends to improve as they move into late adolescence. However, more severe conflicts between adolescents and their parents during the early adolescent phase may yield more profound challenges [14, 15], potentially affecting parents’ HRQOL. For instance, parents may endure distress and heightened stress levels, potentially impacting their HRQOL and caregiving role [6, 16]. Among parents of young children from the general Swedish population, mental health problems were associated with lower parental HRQOL. Additionally, households with at least one individual encountering difficulties and necessitating support evaluated their HRQOL as lower compared with families without such issues [17].

The global public health crisis caused by the COVID-19 pandemic adversely affected the health and overall wellbeing of individuals and societies worldwide. These effects were particularly pronounced in the early stages of the pandemic, when heightened economic and social disruption was associated with increased levels of stress, depression, and anxiety. Clear disparities emerged, with lower HRQOL reported among women, younger individuals, and those with lower socioeconomic status [18–21]. In addition, parents experienced elevated stress, likely due to disruptions in daily routines and social connections [22–24]. In countries such as Norway, the pandemic was managed relatively adeptly through governmental measures aimed at safeguarding employment and providing economic compensations. However, despite this comprehensive response, some Norwegian residents still experienced stress and insecurity as consequences of the pandemic [25, 26]. Over time, mental health trends indicated a degree of adaptation increases in symptoms were generally modest and declined gradually, returning to levels close to those observed before the pandemic by mid-2020 in most population groups. This trajectory underscores both the widespread impact of the pandemic and a notable degree of resilience within the population [18–21].

Longitudinal studies into the potential variations in parents’ HRQOL while their adolescent transition through adolescence appear scarce, with previous studies predominantly centering on parents of adolescents with specific conditions or ailments [6, 27–29]. It is important to assess the HRQOL of parents within the general population, as adolescents often require parental support, particularly amid ongoing crises [6, 15, 30, 31]. The objective of the present study is to describe levels of HRQOL in parents of adolescents over a span of four years (2019, 2021, and 2023) and identify which of selected sociodemographic variables are associated with HRQOL over time. In addition, we compared all HROQL domains with norm data from the general Norwegian population.

## Methods

### Sample and data collection

The present study constitutes a component of the ‘Start Young: Quality of Life and Pain in Generations’ project and expands upon the results derived from our preceding studies into the HRQOL experienced by parents of adolescents [32, 33]. The ‘Start Young: Quality of Life and Pain in Generations’ [34–37] is a Norwegian longitudinal study that focuses on adolescents and their parents. This current research utilised data obtained from the parents at the initial phase, specifically when their adolescent child was between 14 to 15 years (spanning November 2018 to April 2019) [14], alongside data gathered from January to February 2021, at which point the adolescents were aged 16 to 17 years amid the ongoing COVID-19 pandemic, and January to February 2023.

In 2019, one parent of each adolescent aged 14 to 15 years from 22 schools (varying in both size and location) was invited to participate in the study. Potential participants encompassed 1,663 adolescent-parent dyads from the participating schools. A total of 556 parents (33.4%) completed the questionnaire in 2019. All parents who participated in 2019 (*N* = 556) were subsequently sent a text message in 2021 and 2023, which included a link to the survey along with an invitation to participate. Should they fail to complete the second or third survey, they received up to three reminders. In accordance with the approval granted by the Norwegian Centre for Research Data, this was the only permitted method for contacting the parents. In total, 317 parents (response rate 57.0%) completed the survey in 2021, and 231 parents (response rate 41.5%) completed the survey in 2023 and were included in this study.

Data collection transpired via a web-based questionnaire administered at all designated time points. A secure data server was employed for the storage of collected data. Questionnaires from 2019 were linked to those from 2021 to 2023 through the application of participants’ respective ID numbers. All study protocols received approval from the Norwegian Centre for Research Data (Reference: 60981).

All questionnaires had undergone prior translation into Norwegian and validation processes. The questionnaires employing summative scales exhibited satisfactory Cronbach’s alpha values as evidenced in a prior publication [32]. Most questions included a neutral option, ensuring that all items received responses. For the parents, data collection was solely concentrated on aspects related to them, excluding information concerning their child.

### Instruments

The sociodemographic variables and questionnaires for data collection in 2019, 2021 and 2023 were mainly the same.

### Sociodemographic variables

The first part of the questionnaire included self-reported data on demographic variables such as gender, age, marital status, education, household income and geographical region. Possible region-related differences were included in multiple analyses because a previous study showed a negative pattern within some psychosocial variables in one of the regions (i.e., more people being on disability benefits and the use of anti-depressive medication) [38].

### HRQOL

The RAND-36 was used to assess HRQOL. RAND-36 is a generic questionnaire that includes eight domains: general health, bodily pain, physical function, physical role function, mental health, vitality, social function and emotional role function. These domains can be combined into physical and mental sum scales that reflect physical (physical component summary [PCS]) and mental (mental component summary [MCS]) health. The RAND-36 scales were scored according to published scoring procedures, and each scale was transformed to range from 0 to 100, with 100 representing excellent health [39–42]. At baseline, the Cronbach’s α values for the current study were 0.89 for mental health, 0.89 for bodily pain, 0.83 for general health, 0.87 for social function, 0.89 for physical function, 0.93 for physical role function and 0.87 for emotional role function.

Some studies have reported on Norwegian RAND-36 or SF-36 norm data from the general Norwegian population, all of which concluded minor differences in mean and standard deviations (SD) over the years [4, 43, 44]. We chose to use norm data with unadjusted means and SDs, and joints values for women and men, in the age group including the mean age of the parents in the current study [4].

### Statistical analyses

All statistical analyses were conducted using IBM SPSS Statistics for Windows, Version 29.0 (IBM Corp., Armonk, NY, USA) and Stata, version 17. Descriptive statistics were calculated for all variables, and data are presented as means with SDs or as counts and percentages for continuous and categorical variables, as appropriate. Independent-samples *t*-tests were used to compare crude differences in RAND-36 between the parents in the current study and Norwegian norm data [4].

A linear mixed model for repeated measures with an identity link function was used to examine associations between selected sociodemographic variables and changes in HRQOL over the four-year period. The non-independence of repeated measures from the same individual was accounted for using ID as a random factor. We did not impose any parametric structure on our data and used unstructured covariance matrix. Age, gender, marital status (cohabiting/living alone), education, employment status, household income and time (2019, 2021 and 2023) were fitted as fixed factors. These variables were chosen as possible predictive factors because they were statistically significantly associated with HRQOL in 2019 and identified as predictive factors of HRQOL in previous studies. The results are presented as the estimated regression coefficients (B) with 95% confidence intervals (CI). All analyses were considered exploratory, so no correction for multiple testing was made, and p-values < 0.05 were considered significant. All tests were two-sided.

## Results

### Characteristics of the sample

The sociodemographic characteristics of the parents in 2019 are provided in Table 1. In 2019, most of the parents were mothers (77%), married or cohabitating (81%), had a university education of four years or more (51%), worked full time (74%) or part time (17%) and had a household income of more than NOK 1 million (51%). The same patterns were assessed also in 2021 (*n* = 317) and 2023 (*n* = 231) There were some minor differences between responders and non-responders in 2021 and 2023 (se supplementary tables) compared to responders in 2019. More non-responders were in the lowest education-group and reported lower scores within RAND-36 physical function in 2021, and in 2023 non-responders reported lower scores within both the RAND-36 general health and physical function.

**Table 1:** Characteristics of the sample in 2019 (*n* = 556)

Demographic	*n*
Age, years	45 (3)
Mothers	431 (77%)
Fathers	125 (23%)
*Living condition*	
Married/cohabitating	454 (81%)
Single/ Divorced or separated/widow(er)	103 (19%)
*Education*	
Education less than 13 years	134 (24%)
University < 4 years	140 (25%)
University ≥4 years	282 (51%)
*Employment status*	
Full time	411 (74%)
Part time	103 (19%)
Not in paid work	42 (7%)
*County*	
Oslo/Akershus	245 (44%)
Agder	311 (56%)
*Household income (NOK)*	
< 450 000	47 (9%)
450 000–750 000	96 (17%9
751 000–1,000,000	128 (23%)
More than 1,000,000	285 (51%)

Categorical data are presented as number (%) and continuous variables as mean (SD)

### Health-related quality of life

Descriptive characteristics of parents’ HRQOL in 2019, 2021 and 2023 together with the norm values derived from the Norwegian general population and their crude comparisons are presented in Table 2. We identified some differences. The parents in the current study reported significantly higher score for RAND-36 PCS in 2019 (51.7 (8.8), *p* = 0.006) and in 2021 (52.1 (8.5), *p* = 0.024) compared with norm data (50.8 (9.1)). Furthermore, the parents reported significantly lower MCS scores in 2021 ((51.1 (9.6), *p* = 0.014)) and in 2023 (50.7 (10.6), *p* = 0.008) compared with norm data (52.3 (9.1)) [4]. We also identified small differences in some of the eight RAND-36 domains (see Table 2).

**Table 2:** Descriptive characteristics of HRQOL in 2019, 2021 and 2023 compared with Norwegian norms [1](ref)

	2019		2021	2023	Norwegian norms	2019	2021	2023
	*N* = 556		*N* = 317	*N* = 231	*N* = 1054–1080	*p*-values	*p*-values	*p*-values
HRQOL								
RAND-36 PCS^a^	51.7 (8.8)	52.1 (8.5)		51.0 (9.7)	50.8 (9.1)	0.006	0.024	0.788
RAND-36 MCS^a^	52.3 (8.1)	51.1 (9.6)		50.7 (10.6)	52.3 (9.1)	0.930	0.018	0.008
RAND-36 eight domains								
Bodily pain	78.5 (23.3)	78.2 (22.8)		74.5 (24.9)	74.5 (23.4)	0.002	0.021	0.991
General health	76.8 (19.3)	76.1 (19.7)		74.2 (22.0)	78.2 (20.7)	0.189	0.110	0.009
Physical function	93.5 (13.2)	93.4 (13.9)		92.2 (14.7)	90.9 (15.4)	< 0.001	< 0.001	0.223
Physical role lfunction	84.0 (33.1)	83.9 (31.6)		81.0 (36.5)	82.6 (32.8)	0.427	0.544	0.499
Mental health	81.1 (12.5)	79.7 (14.1)		78.6 (15.8)	80.7 (15.6)	0.766	0.712	0.522
Vitality	64.1 (20.0)	63.3 (20.4)		62.4 (22.6)	62.1 (20.1)	0.052	0.337	0.820
Social function	87.1 (19.8)	86.9 (20.1)		83.9 (24.0)	87.7 (20.1)	0.546	0.519	0.011
Emotional role function	89.3 (27.4)	83.4 (33.5)		83.4 (34.0)	89.0 (26.9)	0.854	0.002	0.006

^a^ The score for the RAND-36 ranges from 0 to 100, where 100 indicates a high HRQOL. Data are presented as number (%) and continuous variables as mean (SD)

*PCS* physical component summary, *MCS* mental component summary

### HRQOL over time

When adjusted for gender, age, county, living conditions, level of education, employment status and household income, there were statistically differences over time for most RAND-36 domains, although of limited clinical importance (see Table 3). For the RAND-36 *physical function*, the adjusted scores were statistically significantly lower compared with those of 2019. However, the change was limited in size, less than one point in 2021 compared with 2019 (B=-0.77, 95% CI [–1.86 to − 0.31]). and 2.6 points lower in 2023 compared with 2019, (B=-2.55, 95% CI [–3.78 to − 1.33]). For the RAND-36 *bodily pain*, the adjusted scores were more than four points lower in 2023 compared with 2019 (B = − 4.10, 95% CI [–6.62 to − 1.58]). Also, for *general health*, the adjusted scores were more than four points lower in 2023 compared with 2019 (B = − 4.23, 95% CI [–5.86 to − 2.60]). For the *vitality*, the adjusted scores were nearly three points lower in 2023 compared with 2019 (B = − 2.50, 95% CI [–4.57 to − 0.43]), whereas for *social function* the adjusted scores were almost five points lower in 2023 compared with 2019 (B = − 4.50, 95% CI [–6.76 to − 2.25]). For *emotional role function*, the adjusted scores were more than 6 points lower in 2021 compared with 2019 (B = − 6.34, 95% CI [–10.02 to − 2.65]), and the parents scored 7.1 points lower in 2023 compared with 2019 (B = − 7.14, 95% CI [–11.78 to − 3.01]), also considered clinically important [41, 45]. For *mental health*, the adjusted scores were 1.6 points lower in 2021 compared with 2019 (B=-1.64, 95% CI [–2.98 to − 0.30]) and 3.4 points lower in 2023 compared with 2019 (B = − 3.41, 95% CI [–4.92 to − 1.89]). For the summary scores, *PCS*, the adjusted scores were about one point lower in 2023 compared with 2019 (B = − 0.95, 95% CI [–1.82 to − 0.08]). For MCS the adjusted scores were 1.3 points lower in 2021 compared with 2019 (B = − 1.27, 95% CI [–2.22 to − 0.32]) and 2.1 points lower in 2023 compared with 2019 (B = − 2.14, 95% CI [–3.21 to − 1.07]).

**Table 3:** Mixed model for associations between selected sociodemographic and changes in HRQOL (RAND-36) over a 4-year period (2019, 2021 and 2023)

	Physical function			Physical role function		
	B	(95% CI)	*p*-values	B	(95% CI)	*p*-values
*Time*						
2019 (ref)						
2021	− 0.77	(-1.86, − 0.31)	0.161	-1.27	(-4.89, 2.35)	0.492
2023	-2.55	(-3.78, -1.33)	< 0.001	-3.15	(-7.22, 0.92)	0.129
*Gender* (Ref = father)						
Mothers	-1.15	(-3.53, 1.24)	0.345			
*Age*	− 0.19	(-0.40, 0.02)	0.075	− 0.06	(-0.55, 0.43)	0.806
*County* (ref=Oslo/Viken)						
Agder	-1.07	(-3.21, 1.06)	0.325	-3.75	(-8.60, 1.10)	0.129
*Living conditions* (ref= Married/cohabitat)						
Single/divorced, widow/widower	2.20	(-0.82, 5.21)	0.154	1.99	(-4.91, 8.88)	0.572
*Education* (ref. less than 13 years)						
University less than 4 years	1.17	(-1.71, 4.06)	0.424	4.01	(-2.59, 10.62)	0.234
University 4 years or more	2.85	(0.13, 5.57)	0.040	5.72	(-0.48, 11.93)	0.070
*Employment status* (ref= Full time)						
Part time	− 0.21	(-2.83, 2.39)	0.869	-8.58	(-14,51, -2.65)	0.005
Not paid work	-19.55	(-23.36, -15.74)	< 0.001	-36.10	(-44.73, -27.45)	< 0.001
*Household income* (NOK)(ref < 450.000)						
451.000–750.000	8.99	(4.76, 13.21)	< 0.001	5.23	(-4.51, 14.98)	0.292
751,000–1.000,000	9.79	(5.29, 14.29)	< 0.001	12.84	(2.45, 23.22)	0.015
More than 1.000,000	12.00	(7.43, 16.56)	< 0.001	16.02	(5.47, 26.58)	< 0.001

	Bodily pain			General health		
	B	(95% CI)	*p*-values	B	(95% CI)	*p*-values
*Time*						
2019 (ref)						
2021	− 0.83	(-3.07, 1.40)	0.466	-1.37	(-2.81, 0.07)	0.062
2023	-4.10	(-6.62, -1.58)	0.001	-4.23	(-5.86, -2.60)	< 0.001
*Gender* (Ref = father)						
Mothers	-4.43	(-8.46, -0.41)	0.031	− 0.90	(-4.42, 2.62)	0.617
*Age*	− 0.16	(-0.51, 0.20)	0.398	− 0.13	(-0.44, 0.19)	0.433
*County* (ref=Oslo/Viken)						
Agder	− 0.64	(-4.24, 2.96)	0.727	-2.41	(-5.57, 0.75)	0.135
*Living conditions* (ref= Married/cohabitat)						
Single/divorced, widow/widower	1.61	(-3.48, 6.71)	0.535	-2.40	(-6.86, 2.05)	0.290
*Education* (ref. less than 13 years)						
University less than 4 years	2.80	(-2.08, 7.67)	0.261	-1.80	(-6.05, 2.46))	0.408
University 4 years or more	4.08	(-0.51, 8.67)	0.081	1.01	(-3.01, 5.02)	0.623
*Employment status* (ref= Full time)						
Part time	-4.23	(-8.63, 0.17)	0.059	-6.62	(-10.48, -2.77)	0.001
Not paid work	-27.76	(-34.18, -21.33)	< 0.001	-25.62	(-31.26, 19.98)	< 0.001
*Household income* (NOK)(ref < 450.000)						
451.000—750,000	5.37	(-1.79, 12.53)	0.142	5.91	(-0.32, 12.15)	0.063
751.000–1.000,000	7.66	(0.02, 15.30)	0.049	4.30	(-2.34, 10.94)	0.205
More than 1.000,000	12.24	(4.49, 19.99)	0.002	7.94	(1.20, 14.67)	0.021

	Vitality			Social function		
	B	(95% CI)	*p*-values	B	(95% CI)	*p*-values
*Time*						
2019 (ref)						
2021	− 0.91	(-2.75, 0.93)	0.331	− 0.50	(-2.50, 1.50)	0.624
2023	-2.50	(-4.57, -0.43)	0.018	-4.50	(-6.76, -2.25)	< 0.001
*Gender* (Ref = father)						
Mothers	-4.02	(-7.57, -0.47)	0.026	-4.55	(-8.05, -1.04)	0.011
*Age*	0.21	(-0.11, 0.52)	0.200	− 0.04	(-0.35, 0.27)	0.801
*County* (ref=Oslo/Viken)						
Agder	-1.90	(-5.08, 1.28)	0.241	-1.38	(-4.50, 1.75)	0.389
*Living conditions* (ref= Married/cohabitat)						
Single/divorced, widow/widower	-3.63	(-8.12, 0.86)	0.113	-3.90	(-8.33, 0.53)	0.085
*Education* (ref. less than 13 years)						
University less than 4 years	0.86	(-3.43, 5.16)	0.694	0.37	(-3.87, 4.61)	0.864
University 4 years or more	1.19	(-2.85, 5.24)	0.563	0.24	(-3.75, 4.22)	0.908
*Employment status* (ref= Full time)						
Part time	-3.81	(-7.69, 0.07)	0.054	-3.32	(-7.14, 0.50)	0.067
Not paid work	-24.79	(-30.46, -19.12)	< 0.001	-23.88	(-29.46, 18.29)	< 0.001
*Household income* (NOK) (ref < 450.000)						
451,000–750,000	0.77	(-5.53, 7.08)	0.811	5.83	(-0.40, 12.07)	0.067
751,000–1,000,000	3.74	(-2.99, 10.46)	0.276	10.74	(4.10, 17.39)	0.002
More than 1,000,000	6.40	(-0.42, 13.23)	0.066	12.25	(5.50, 19.00)	< 0.001

	Emotional role function			Mental health		
	B	(95% CI)	*p*-values	B	(95% CI)	*p*-values
*Time*						
2019 (ref)						
2021	-6.34	(-10.02, -2.65)	0.001	-1.64	(-2.98, − 0.30)	0.017
2023	-7.14	(-11.28, 3.01)	0.001	-3.41	(-4.92, -1.89)	< 0.001
*Gender* (Ref = father)						
Mother	-5.97	(-0.11.09, − 0.85)	0.022	-1.87	(-4.34, 0.59)	0.136
*Age*	0.25	(-0.21, 0.71)	0.291	0.25	(0.02, 0.47)	0.029
*County* (ref=Oslo/Viken)						
Agder	-1.09	(-5.63, 3.45)	0.638	1.75	(-0.45, 3.96)	0.119
*Living conditions* (ref= Married/cohabitat)						
Single/divorced, widow/widower	-1.86	(-8.34, 4.62)	0.573	-2.60	(-5.71, 0.52)	0.102
*Education* (ref. less than 13 years)						
University less than 4 years	2.63	(-3.58, 8.85)	0.406	− 0.57	(-3.56, 2.41)	0.705
University 4 years or more	2.49	(-3.33, 8.31)	0.402	− 0.92	(-3.73, 1.88)	0.519
*Employment status* (ref= Full time)						
Part time	-2.71	(-8.28, 2.85)	0.340	-2.16	(-4.85, 0.53)	0.115
Not paid work	-22.12	(-30.23, 14.02)	< 0.001	-9.36	(-13.28, -5.42)	< 0.001
*Household income* (NOK) (ref < 450.000)						
451,000–750,000	-2.13	(-11.31, 7.05)	0.649	-2.12	(-6.50, 2.26)	0.342
751,000–1,000,000	6.49	(-3.30, 16.28)	0.194	1.33	(-3.34, 6.00)	0.576
More than 1,000,000	6.21	(-3.74, 16.17)	0.221	3.59	(-1.15, 8.33)	0.137

	PCS			MCS		
	B	(95% CI)	*p*-values	B	(95% CI)	*p*-values
*Time*						
2019 ( ref)						
2021	− 0.01	(-0.78, 0.76)	0.982	-1.27	(-2.22, -0.32)	0.009
2023	− 0.95	(-1.82, -0.08)	0.033	-2.14	(-3.21, -1.07)	< 0.001
*Gender* (Ref = father)						
Mother	− 0.71	(-2.22, 0.80)	0.356	-1.87	(-3.46, − 0.27)	0.021
*Age*	− 0.12	(-0.26, 0.02)	0.082	0.14	(-00, 0.29)	0.050
*County* (ref=Oslo/Viken)						
Agder	-1.18	(-2.53, 0.17)	0.087	0.23	(-1.19, 1.64)	0.754
*Living conditions* (ref= Married/cohabitat)						
Single/divorced, widow/widower	0.91	(-1.01, 2.82)	0.352	-2.18	(-4.19, -0.16)	0.034
*Education* (ref. less than 13 years)						
University less than 4 years	0.69	(-1.14, 2.52)	0.461	− 0.02	(-2.00, 1.91)	0.982
University 4 years or more	1.77	(0.04, 3.49)	0.044	− 0.45	(-2.27, 1.36)	0.624
*Employment status* (ref= Full time)						
Part time	-2.03	(-3.68, -0.38)	0.016	-1.21	(-2.95, 0.53)	0.173
Not paid work	-12.06	(-14.47, -9.64)	< 0.001	-6.61	(-9.15, -4.08)	< 0.001
*Household income* (NOK) (ref < 450.000)						
451,000–750,000	3.95	(1.27, 6.64)	0.004	-1.39	(-4.23, 1.45)	0.336
751.000–1,000,000	4.16	(1.30, 7.02)	0.004	1.15	(-1.88, 4.18)	0.457
More than 1,000,000	5.67	(2.77, 8.58)	< 0.001	1.63	(-1.44, 4.71)	0.299

^a^ The score for the RAND-36 ranges from 0 to 100, where 100 indicates a high HRQOL

PCS, physical component summary; MCS, mental component summary

The results are presented as the estimated regression coefficients (B) with 95% confidence intervals (CI). All the analyses were considered exploratory, so no correction for multiple testing was done, and p-values < 0.05 were considered significant. All tests were two-sided

1. Garratt AM, Stavem K. Measurement properties and normative data for the Norwegian SF-36: results from a general population survey. Health Qual Life Outcomes. 2017;15(1):51. doi: 10.1186/s12955-017-0625-9

### Sociodemographic characteristics associated with levels of HRQOL

We identified which of selected sociodemographic variables were associated with HRQOL over time, measured by RAND-36. All models were adjusted for gender, age, county, living conditions, level of education, employment status and household income (see Table 3). County was not statistically significantly associated with HRQOL (all *p* > 0.05; see Table 3).

#### Physical function

Regarding education, parents who had more than 4 years of university education had almost 3 points higher level of *physical function* compared with those with the lowest education (B = 2.85; 95% CI [0.13–5.57]) (not clinically relevant). However, there were no differences in reported physical function for those with the lowest and middle education. Parents who were not in paid work scored almost 20 points lower than those in full-time paid work (B = − 19.55, 95% CI [–23.36 to -15.74]). The most important predictive factor for levels of physical function was household income. Those who reported having an income of over NOK 450.000 scored all significantly higher than those earning less than this income level. The highest effect was observed for those whose household income was over NOK 1,000,000; they scored 12 points higher than those with the lowest household income (B = 12.00, 95% CI [7.43 to 16.56]).

#### Physical role function

Parents who were not in paid work scored 36 points lower than those who were in full-time paid work (B = − 36.10, 95% CI [–44.73 to − 27.45]) for *physical role function*, while those who worked part time scored nearly nine points lower (B = − 8.58, 95% CI [–14.51 to − 2.65]). Parents who reported an income of over NOK 450.000 all scored significantly higher than those earning less than this income level. The highest effect was observed for those whose household income was over NOK 1,000,000, they scored 16 points higher compared with those with the lowest household income (B = 16.02, 95% CI [5.47 to 26.58]).

### Bodily pain

Mothers had more than 4 points lower *bodily pain* score compared with fathers (B = − 4.43; 95% CI [–8.46 to − 0.41]) (not clinically relevant). Parents who were not in paid work scored almost 28 points lower on bodily pain compared with those in full-time paid work (B = − 27.76, 95% CI [–34.18 to − 21.33]). Furthermore, for household income those who reported having an income of over NOK 450.000 all scored significantly higher on bodily pain than those earning less than this income level. The highest effect was observed for those whose household income was over NOK 1,000,000NOK; they scored 12 points higher compared with those with the lowest household income (B = 12.24, 95% CI [4.49 to 19.99]).

### General health

Parents who were not in paid work scored almost 26 points lower than those in full-time paid work (B = − 25.62, 95% CI [–31.26 to − 19.98]) for *general health*, while those who worked part time scored almost 7 points lower scores (B = − 6.62, 95% CI [–10.48 to − 2.77]). Furthermore, parents with household income over NOK 1,000,000 scored almost 8 points higher compared with those with the lowest household income (B = 7.94, 95% CI [1.20 to 14.67]).

### Vitality

Mothers had more than 4 points lower *vitality* score than fathers (B = − 4.04; 95% CI [–7.57 to − 0.47]) (not clinically relevant), and parents who were not in paid work scored almost 25 points lower than those in full-time paid work (B = − 24.79, 95% CI [–30.46 to − 19.12]).

### Social function

Also, for *social function* mothers reported more than 4 points lower than fathers (B = − 4.55; 95% CI [–8.05 to − 1.04]) (not clinically relevant), and parents who were not in paid work scored almost 24 points lower compared with those in full-time paid work (B = − 23.88, 95% CI [–29.46 to − 18.29]).

Furthermore, for household income, those who reported having an income of over NOK 450.000 scored all significantly higher than those earning less. The highest effect was observed for those whose household income was over NOK 1,000,000. They scored 12 points higher than those with the lowest household income (B = 12.25, 95% CI [5.50 to 19.00]).

### Emotional role function

Mothers reported almost 6 points lower score compared with fathers (B = − 5.97; 95% CI [–11.09 to − 0.85]) for *emotional role function*, and parents who were not in paid work scored 22 points lower than those in full-time paid work (B = − 22.12, 95% CI [–30.23 to − 14.02]).

### Mental health

Parents who were older reported 0.25 points higher for *mental health* (B = 0.25, 95% CI [0.02 to 0.47]) (not clinically relevant), and parents who were not in paid work scored more than 9 points lower than those in full-time paid work (B = − 9.36, 95% CI [–13.28 to − 5.42]).

### Summary scores

For *PCS*, parents with university education of 4 years or more reported a nearly 2 points higher than those with less education (B = 1.77; 95% CI [0.04 to 3.49]) (not clinically relevant), and parents who were not in paid work scored 12 points lower compared with those in full-time paid work (B = − 12.06, 95% CI [–14.47 to − 9.64]). Furthermore, for household income, those who reported having an income of over NOK 450.000 all scored significantly higher than those earning less. The highest effect was observed for those whose household income was over NOK 1,000,000. They scored nearly 6 points higher than those with the lowest household income (B = 5.67, 95% CI [2.77 to 8.58]). Mothers scored nearly 2 points lower than fathers (B = − 1.87; 95% CI [–3.46 to − 0.27]) (not clinically relevant) for *MCS*; parents who were living without a partner scored 2.2 points lower than those living with a partner (B = − 2.18; 95% CI [–4.19 to − 0.16]) (not clinically relevant), and parents who were not in paid work scored 6.6 points lower than those in full-time paid work (B = − 6.61, 95% CI [–9.15 to − 4.08]).

## Discussion

During the four-year period from 2019 to 2023, we observed a small but statistically significant decline in most of the HRQOL domains, and no domains increased again after the COVID-19 pandemic. Higher education, being in paid work and having a high household income were the most important associates of high HRQOL scores. We identified some statistical differences in HRQOL between the parents in the current study points and Norwegian norms for the general population at all time points. However, they were of limited clinical relevance.

For four years, parents’ HRQOL exhibited a modest but persistent decline in mental domains, without evidence of rebound by 2023, while physical summary scores remained comparatively stable. When interpreted against normative data, these small average differences may appear modest [1, 40, 41, 46]. The findings align with international studies documenting sustained mental health challenges during and after the COVID-19 pandemic [25, 47, 48]. Comparing with Norwegian SF/RAND-36 norms further underscores the importance of these deviations [4, 44].

Socio-economic gradients were consistent and more pronounced for physical than mental HRQOL domains. Higher household income and education were associated with better HRQOL across most domains, with the steepest associations for physical functioning, physical role function, general health, bodily pain and PCS. For example, parents with a household income higher than NOK 1,000,000 scored 16 points higher on physical role function than those at the lowest income level. In mental health, a higher income was associated with nearly six-point higher scores, while for social function this difference was 12 points. This pattern aligns with evidence that economical and educational resources more directly influence physical domains through safer work and living conditions, access to care and rehabilitation, and capacity to manage pain and functional limitation, whereas mental health reflects broader psychosocial exposures that attenuate SES gradients [2, 5, 49, 50]. The positive, albeit smaller, association between four or more years of university education and PCS is consistent with roles for health literacy and self-management in preserving physical functioning [51–54].

Employment status emerged as a strong correlation of HRQOL across domains, with particularly large differences for role limitations. Relative to full-time paid work, not being in paid work was associated with substantially lower scores on physical role function and emotional role function; part-time work showed intermediate differences. Similarly, though on a smaller scale, gaps were observed for mental health. These patterns are compatible with both causal benefits of employment (structure, income, social integration) and health-selection mechanisms (‘healthy worker’ effects), a duality well documented in HRQOL and labour health research [53–55]. Furthermore, gender differences were modest but consistent throughout the years. Mothers reported lower scores than fathers on emotional role function. Several mechanisms likely contributed to this, including unequal distribution of unpaid caregiving and household management, the mental load of coordinating family life, and gender differences in job security or flexibility that intensify role conflict and emotional strain [16, 56].

Bodily pain emerged as one of the HRQOL domains with the clearest gender and socio-economic gradients in this study. Mothers reported more pain than fathers did. Although not considered clinically relevant, this finding that aligns with previous research showing that women report a higher prevalence and severity of pain than men across the life course [6, 32, 57]. Gender differences in pain may reflect a combination of biological factors as well as psychosocial mechanisms, including higher cumulative caregiving demands, greater emotional labour, and increased exposure to chronic stressors [58]. Among parents of adolescents, pain may further interact with mental load, sleep disruption and emotional strain, potentially amplifying its impact on overall HRQOL.

Employment status also showed a particularly strong association with bodily pain. Parents who were not in paid work reported substantially lower pain scores—nearly 28 points lower than those in full-time paid work—representing a difference that is clearly clinically meaningful. This finding is consistent with earlier studies demonstrating close links between pain, reduced work ability and exclusion from the labour market [32, 57]. Pain may contribute to withdrawal from paid work, while being outside employment may in turn worsen pain through reduced physical activity, social participation, income insecurity and loss of daily structure, suggesting bidirectional and self-reinforcing mechanisms [17].

The well-being of parents is important to the parents themselves; however, it is also a key determinant of adolescents’ life conditions and psychosocial development. Parental mental and emotional health influences parenting practices, family climate and the availability of emotional support, all of which shape adolescents developmental trajectories [59, 60]. Supporting parents of adolescents requires coordinated public health and governmental strategies that address both mental and physical health needs. Accessible mental health services, including low-threshold counseling and digital interventions, can help mitigate persistent declines in mental health observed in this population [11]. Parenting programs that strengthen coping skills and promote health literacy are essential, particularly for families with lower socio-economic resources [51]. Governmental policies such as flexible work arrangements and income support can reduce role strain and improve overall HRQOL, while integrated family support systems can address complex needs across health, education and social domains. Community-based approaches, including peer support networks and school–parent partnerships, may further enhance resilience and reduce disparities. These strategies align with global adolescent health frameworks and Norwegian public health priorities, emphasising equity and long-term well-being for families [11, 25].

### Methodological considerations

The longitudinal nature of the study is a strength. We planned and began the four-year follow-up Start Young study before the COVID-19 pandemic hit, and were able to collect data before, during and after the pandemic. Furthermore, the number of parents reported at all time points were relatively high despite the attrition rate. When comparing those who dropped out with those still in the study in 2023, we identified small differences between the groups [33]. See Supplementary files for more details. The questions and questionnaires used in the current study were all well validated. Limitations of the study are the low response rate. Unfortunately, such low response rates are common in studies of the general population [61, 62]. The parents in the present study might be considered unrepresentative of the general parents of adolescents. Participants were mostly mothers, highly educated, married or cohabiting, in paid work parents. Despite this, they report HRQOL scores comparable to previous Norwegian norm data for the age group [4].

## Conclusion

In this four-year longitudinal study, parents of adolescents experienced a small but persistent decline in HRQOL, particularly in mental health domains, from before the COVID-19 pandemic through 2023. Notably, HRQOL did not return to pre-pandemic levels, indicating a lack of full recovery within the study period. Socio-economic factors were consistently associated with HRQOL with higher education, higher household income and participation in paid work linked to better HRQOL. These findings highlight parents of adolescents as an important target group for health promotion and preventive policies. Given parents’ central role in adolescents’ health and development, policies that promote parental HRQOL should be considered a key component of adolescent and family health strategies.

## Supplementary Information

Supplementary Material 1.

## Data Availability

The datasets used and/or analyzed during the current study are not publicly available due to General Data Protection Regulation laws but are available from the corresponding author on reasonable request and with permission from the Norwegian Centre for Research Data.

## Abbreviations

HRQOL: health-related quality of life
PCS: physical component summary score
MCS: mental component summary score
