# Supplementary material for: Health-related quality of life in parents of adolescents in 2019, 2021 and 2023
Source: BMC Pediatr. 2026 Jun 15;26:783. doi: 10.1186/s12887-026-07115-8 (PMC13508395; doi:10.1186/s12887-026-07115-8)
Supplement: Supplementary file 1 [file 12887_2026_7115_MOESM1_ESM.docx]

**Table 1** Baseline characteristics of the non-responders (n=252) versus responders (n=309) at 2-year follow-up

| Demographic | Non-responders N=252 | Responders N=309 | p-value |
| --- | --- | --- | --- |
| Mothers | 184 (73%) | 252 (82%) | 0.016* |
| Fathers | 68 (27%) | 57 (18%) |  |
| Age, years mean (SD) | 45.04 (5.26) | 45.54 (4.36) | 0.070 |
| *Living condition* |  |  | 0.382 |
| Married/cohabitating | 208 (83%) | 249 (81%) |  |
| Single | 16 (6%) | 17(5%) |  |
| Divorced or separated | 24 (9%) | 31 (13%) |  |
| Widowed | 4 (2%) | 2 (1%) |  |
| *Education* |  |  | <0.001* |
| Education less than 13 years | 79 (31%) | 58 (19%) |  |
| University <4 years | 66 (26%) | 75 (34%) |  |
| University ≥4 years | 107 (43%) | 176 (57%) |  |
| *Employment status* |  |  | 0.444 |
| Full time | 181 (72%) | 233 (75% |  |
| Part-time | 53 (21%) | 52 (17%) |  |
| Not working | 18 (7%) | 24 (8%) |  |
| *Household income (NOK)* |  |  | 0.376 |
| <250,000 | 4 (2%) | 1 |  |
| 250,000–450,000 | 23 (9%) | 20 (7%) |  |
| 451,000–750,000 | 44 (17%) | 52 (17%) |  |
| 751,000–1,000,000 | 57 (23%) | 72 (23%) |  |
| >1,000,000 | 124 (49%) | 164 (53%) |  |

Categorical data are presented as number (%) and continuous variables as mean (SD).

Chi-square-tests were used to compare differences in categorical variables and independent sample *t* tests for continuous data

* P ≤ 0.05

**Table 2:** Descriptive characteristics of HRQOL, of the non-responders (n=252) versus responders (n=309) at 2-year follow-up

|  | Non-responders N=252 | Responders N=309 | p-values |
| --- | --- | --- | --- |
| HRQOL |  |  |  |
| RAND-36 PCS^a^ | 51.3 (9.2) | 52.0 (8.6) | 0.494 |
| RAND-36 MCS^a^ | 52.2 (8.2) | 52.2 (8.2) | 0.878 |
| RAND-36 eight domains |  |  |  |
| Bodily pain | 77.8 (24.3) | 79.1 (22.4) | 0.443 |
| General health | 76.1 (19.0) | 77.2 (19.9) | 0.495 |
| Physical function | 92.5 (14.1) | 94.1 (12.6) | 0.043* |
| Physical role function | 82.8 (34.6) | 84.9 (32.0) | 0.189 |
| Mental health | 80.9 (13.2) | 81.2 (12.2) | 0.564 |
| Vitality | 64.0 (20.5) | 63.7 (20.1) | 0.947 |
| Social function | 86.5 (20.6) | 87.3 (19.4) | 0.246 |
| Emotional role function | 89.0 (28.4) | 89.4 (26.9) | 0.654 |

Independent samples *t-* tests were used to compare non-responders and responders.

^a^ The score for the SF-36 ranges from 0 to 100, where 100 indicates a high HRQOL. PCS, physical component summary; MCS, mental component summary.

* P ≤ 0.05

**Table 3** Baseline characteristics of the non-responders (n=325) versus responders (n=231) at 4-year follow-up

| Demographic | Non-responders N=325 | Responders N=231 | p-value |
| --- | --- | --- | --- |
| *Gender* |  |  | 0.068 |
| Mothers | 243 (75%) | 188 (81\|%) |  |
| Fathers | 82 (25%) | 43 (19%) |  |
| Age, mean (SD) | 45.40 (5.15) | 45.24 (4.45) | 0.700 |
| *Living condition* |  |  | 0.192 |
| Married/cohabitating | 263 (81%) | 191 (83%) |  |
| Single/ Divorced or separated/widow(er) | 63 (19%) | 40 (17%) |  |
| *Education* |  |  | <0.026* |
| Education less than 13 years | 86 (26%) | 48 (21%) |  |
| University <4 years | 97 (30%) | 43 (19%) |  |
| University ≥4 years | 142 (44%) | 140 (60%) |  |
| *Employment status* |  |  | 0.444 |
| Full time | 234 (72%) | 177 (77%) |  |
| Part-time | 68 (21%) | 35 (15%) |  |
| Not in paid work | 23 (7%) | 19 (8%) |  |
| *Household income (NOK)* |  |  | 0.079 |
| <450 000 | 34 (10%) | 13 (6%) |  |
| 450,000–750,000 | 59 (18%) | 37 (16%) |  |
| 751,000–1,000,000 | 81 (25%) | 47 (20%) |  |
| More than 1,000,000 | 151 (47%) | 134 (58%) |  |

Categorical data are presented as number (%) and continuous variables as mean (SD).

Chi-square-tests were used to compare differences in categorical variables and independent sample *t* tests for continuous data

* P ≤ 0.05

**Table42** Descriptive characteristics of HRQOL of the non-responders (n=325) versus responders (n=231) at

4-year follow-up

|  | Non-responders N=325 | Responders  N=231 | p-values |
| --- | --- | --- | --- |
| HRQOL |  |  |  |
| RAND-36 PCS^a^ | 51.5 (8.9) | 52.0 (8.7) | 0.418 |
| RAND-36 MCS^a^ | 51.9 (8.7) | 53.0 (7.1) | 0.098 |
| RAND-36 eight domains |  |  |  |
| Bodily pain | 78.4 (23.3) | 78.6 (23.3) | 0.915 |
| General health | 74.9 (19.2) | 79.6 (19.1) | 0.004* |
| Physical function | 92.5 (14.8) | 94.8 (10.5) | 0.042* |
| Physical role limitation | 84.3 (32.9) | 83.6 (33.5) | 0.810 |
| Mental health | 80.4 (13.5) | 82.1 (10.9) | 0.098 |
| Vitality | 63.3 (19.5) | 65.3 (20.7) | 0.250 |
| Social function | 86.1 (20.7) | 88.7 (18.3) | 0.125 |
| Emotional role limitation | 88.2 (29.7) | 91.0 (24.4) | 0.218 |

Independent samples *t-* tests were used to compare non-responders and responders.

^a^ The score for the RAND-36 ranges from 0 to 100, where 100 indicates a high HRQOL. Data are presented as number (%) and continuous variables as mean (SD).

PCS, physical component summary; MCS, mental component summary.

* P ≤ 0.05
